# The Immune Checkpoint BTLA in Oral Cancer: Expression Analysis and Its Correlation to Other Immune Modulators

**DOI:** 10.3390/ijms25126601

**Published:** 2024-06-15

**Authors:** Jutta Ries, Leah Trumet, Alina Hahn, Lina Kunater, Rainer Lutz, Carol Geppert, Marco Kesting, Manuel Weber

**Affiliations:** 1 Department of Oral and Cranio-Maxillofacial Surgery, Friedrich-Alexander-Universität Erlangen-Nürnberg (FAU), 91054 Erlangen, Germany; alinamarie.hahn@gmail.com (A.H.); lina.kunater@gmx.at (L.K.); rainer.lutz@uk-erlangen.de (R.L.); marco.kesting@uk-erlangen.de (M.K.); manuel.weber@uk-erlangen.de (M.W.); 2 Deutsches Zentrum Immuntherapie (DZI) and Comprehensive Cancer Center Erlangen-EMN (CCC ER-EMN), Friedrich-Alexander-Universität Erlangen-Nürnberg (FAU), 91054 Erlangen, Germany; leah.trumet@uk-erlangen.de; 3 Department of Operative Dentistry and Periodontology, Friedrich-Alexander-Universität Erlangen-Nürnberg (FAU), 91054 Erlangen, Germany; 4 Institute of Pathology, Friedrich-Alexander University Erlangen-Nürnberg (FAU), 91054 Erlangen, Germany

**Keywords:** BTLA, immune checkpoints, immune therapy, OSCC, oral squamous cell carcinoma, CD96, PD-L1, PD-L2, PD1

## Abstract

In oral squamous cell carcinoma (OSCC) tissues, an immunotolerant situation triggered by immune checkpoints (ICPs) can be observed. Immune checkpoint inhibitors (ICIs) against the PD1/PD-L axis are used with impressive success. However, the response rate is low and the development of acquired resistance to ICI treatment can be observed. Therefore, new treatment strategies especially involving immunological combination therapies need to be developed. The novel negative immune checkpoint BTLA has been suggested as a potential biomarker and target for antibody-based immunotherapy. Moreover, improved response rates could be displayed for tumor patients when antibodies directed against BTLA were used in combination with anti-PD1/PD-L1 therapies. The aim of the study was to check whether the immune checkpoint BTLA is overexpressed in OSCC tissues compared to healthy oral mucosa (NOM) and could be a potential diagnostic biomarker and immunological target in OSCC. In addition, correlation analyses with the expression of other checkpoints should clarify more precisely whether combination therapies are potentially useful for the treatment of OSCC. A total of 207 tissue samples divided into 2 groups were included in the study. The test group comprised 102 tissue samples of OSCC. Oral mucosal tissue from 105 healthy volunteers (NOM) served as the control group. The expression of two isoforms of BTLA (BTLA-1/2), as well as PD1, PD-L1/2 and CD96 was analyzed by RT-qPCR. Additionally, BTLA and CD96 proteins were detected by IHC. Expression levels were compared between the two groups, the relative differences were calculated, and statistical relevance was determined. Furthermore, the expression rates of the immune checkpoints were correlated to each other. BTLA expression was significantly increased in OSCC compared to NOM (pBTLA_1 = 0.003; pBTLA_2 = 0.0001, pIHC = 0.003). The expression of PD1, its ligands PD-L1 and PD-L2, as well as CD96, were also significantly increased in OSCC (*p* ≤ 0.001). There was a strong positive correlation between BTLA expression and that of the other checkpoints (*p* < 0.001; ρ ≥ 0.5). BTLA is overexpressed in OSCC and appears to be a relevant local immune checkpoint in OSCC. Thus, antibodies directed against BTLA could be potential candidates for immunotherapies, especially in combination with ICI against the PD1/PD-L axis and CD96.

## 1. Introduction

Oral cancer, 90% of which originates from the squamous epithelium (OSCC), is one of the most common malignancies in the world. In the year 2020, GLOBOCAN estimated around 377,713 total cases and 177,757 deaths worldwide [1] and the American Cancer Society has recently estimated the incidence of OSCC, with around 54,000 new cases and about 11,580 deaths in the year 2023 alone [2]. Despite improvements in therapies, OSCC has a poor survival rate of about 50% due to detection of the disease in late clinical stages and the development of lymph node metastases, local recurrences and secondary primary tumors. Hence, the development of better diagnostics and therapy is urgently needed [3].

A large number of immune checkpoints regulate the immune response in a finely tuned manner. There are stimulatory factors that enhance the immune response, such as the co-stimulatory checkpoint molecule CD28, and a number of immunosuppressive factors that prevent the destruction of healthy tissue by limiting a sustained immune response. Examples of inhibitory immune checkpoints include PD1, PD-L ligands, CTLA-4, CD96, BTLA. They inhibit T cell activation, T cell function and promote apoptosis, leading to T cell exhaustion. The amplitude and quality of the T-cell response is regulated by a balance between activating and inhibitory signals. All of these regulators can be used in diagnostic and immunotherapeutic approaches.

**Figure 1:**
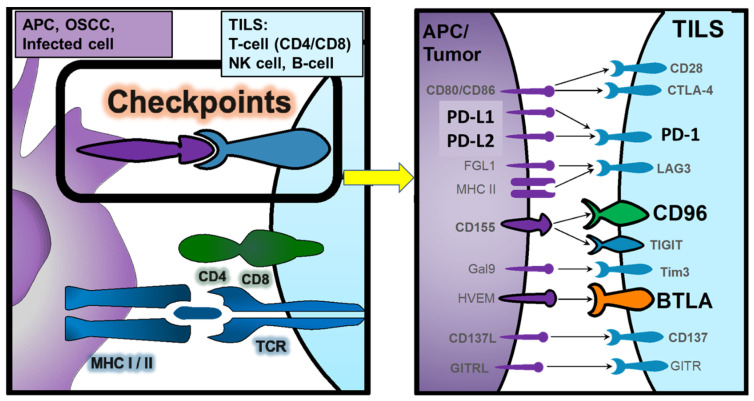
Overview of the analyzed checkpoint molecules (PD1, PD-L1/L2, CD96, BTLA), their interactions to regulate the immune response and the cell expressing them. APC = Antigen presenting cell (monocyte, macrophage, dendritic cell, Langerhans cell, B-cell); OSCC = Oral Squamous Carcinoma Cell, TILS = Tumor-Infiltrating Lymphocytes (T-cell, B-cell); NK = Natural Killer cell.

An overview of the analyzed checkpoint molecules (PD1, PD-L, CD96, BTLA), their interactions to regulate the immune response and the cell expressing them is given in Figure 1.

Immunotherapies are a very promising approach in recent years. Particularly with the development of immune checkpoint inhibitors (ICIs) targeting the PD1/PD-L1 axis. These therapy options have proven to be effective in many types of cancer by stimulating the immune system to attack tumor cells. However, there are also challenges such as primary and acquired resistance that can limit the effectiveness of these therapies. Therefore, the search for diagnostic markers that can predict the response to therapy and the development of new approaches basing on new ICI and combination therapies is gaining importance. Also, for head-and-neck squamous cell carcinomas (HNSCC), including OSCC, immune checkpoint inhibitor (ICI) therapy is increasingly important as it becomes a standard treatment in advanced cases without curative surgical and radiation oncological options [4,5]. Since 2019, Pembrolizumab—an anti-PD1 ICI—is approved for first-line treatment in PD-L1 expressing HNSCC [5]. However, the efficacy of currently available anti-PD1 ICI therapy in HNSCC is 17% of patients responding to therapy and below 30% of patients alive 4 years after therapy due to primary and achieved therapy resistance [5].

One way to address the mechanisms of resistance to ICI therapy and increase its efficacy may be to focus on combination therapy [5]. For this purpose, anti-CTLA-4 in combination with anti-PD-L1 or anti-PD1 ICI therapy has already been tested in clinical trials [5,6]. It was shown that the combination of anti-PD1 and anti-CTLA-4 in the treatment of malignant tumors is a viable strategy with improved efficacy and acceptable side effects. In addition, anti-CTLA-4 treatment may reduce the risk of resistance to anti-PD1 in some patients with low PD-L1 expression and show a synergistic anti-tumor effect [7]. However, larger phase III studies could not improve the superiority of a combination anti-PD1/PD-L1 and anti-CTLA-4 therapy in HNSCC [8,9].

To identify potential further candidates for ICI combinations, we analyzed the expression levels of multiple immune regulatory checkpoints in a Nano String mRNA analysis [10]. Comparing OSCC tissue and healthy oral tissue we found that, among other biomarkers, BTLA, CD96 and PD1 levels were elevated in OSCC [10]. To be more specific, mRNA expression of BTLA was found to be upregulated by a factor of 2.4 in oral cancer compared to healthy mucosa, without reaching statistical significance [10].

Our recently published study revealed differences in expression of CD96 in OSCC compared to healthy controls on mRNA- and protein-level [11]. In tissue, there was a highly significant overexpression of CD96 in OSCC patients compared to the control group [11]. Interestingly, a positive correlation at the mRNA level could be measured between CD96 and PD1, motivating further analyses of potential combination partners in association with OSCC [11].

The B and T lymphocyte attenuator (BTLA, CD272) is similar in structure and function to the immunosuppressive PD1 and CTLA-4 receptors [12]. Additionally, BTLA is mainly expressed on T- and B-lymphocytes, dendritic cells and macrophages and mediates immunosuppression by inhibiting the proliferation and function of T- and B-cells [6,12]. The ligand of BTLA is herpesvirus entry mediator (HVEM, official name TNFRSF14) [6,12]. In the context of malignancies, BTLA is mainly considered as an immunosuppressive checkpoint [12]. In solid and hematological malignancies, dysregulation of HVEM/BTLA is associated with poor prognosis [6]. Overexpression of BTLA for example was correlated with lower overall survival in colorectal cancer, whereas high expression of HVEM was associated with poor prognosis in breast cancer, gastric cancer and metastatic melanoma [6].

BTLA expression seems to be regulated differently in various tumor entities [12]. For example, in colorectal carcinomas, decreased BTLA expression compared to healthy tissue has been demonstrated [12]. In HNSCC, BTLA expression was shown to be significantly decreased in tumor-infiltrating T-cells compared with circulating T-cells [13].

The role of BTLA as therapeutic immune checkpoint is currently tested in phase I and II studies as monotherapy and in combination with PD1-inhibition in advanced solid malignancies and lymphomas [14]. Especially, the combination approaches with PD1 are promising [6,15,16,17]. The synergistic effect of PD1 and BTLA inhibition for glioblastoma and OSCC was also demonstrated in mouse models [18,19].

The aim of the current study was to analyze and compare expression levels of BTLA in OSCC patients and healthy controls and to correlate BTLA expression with other checkpoints including PD1, PD-L1 and CD96. BTLA expression in tissue should give us more information about immune parameters of the tumor microenvironment (TME) as ICI response rates strongly depend on the characteristics of the TME [20].

## 2. Results

The Shapiro–Wilk test showed that there was no normal distribution of the ∆CT values and the labelling indices (ILs) of BTLA within the groups (*p* ≤ 0.05). Therefore, only non-parametric tests were used in statistical analyses.

### 2.1. Demographic, Clinical and Histomorphologic Characteristics of the Study Groups

In total 207 subjects, divided into 2 groups, a test group (OSCC) and a control group (NOM) were included in the study. The age and gender of all study participants were documented and are shown in Table 1. The two groups of the collective were matched with regard to gender (*p* = 0.27), but not age (*p* = 0.01). Additionally, the parameters for OSCC staging were determined and are displayed in Table 1.

### 2.2. Comparison of BTLA Expression in Tissue between OSCC and NOM Group

BTLA expression data were derived either from RT-qPCR and are presented as ΔCT values or as labelling indices if generated by IHC. In RT-qPCR the expression of two transcript variants were analyzed. Expression data were not normally distributed, Shapiro–Wilk testing revealed for both variants a *p*-value ≥ 0.05.

### 2.3. RT-qPCR

**Figure 2:**
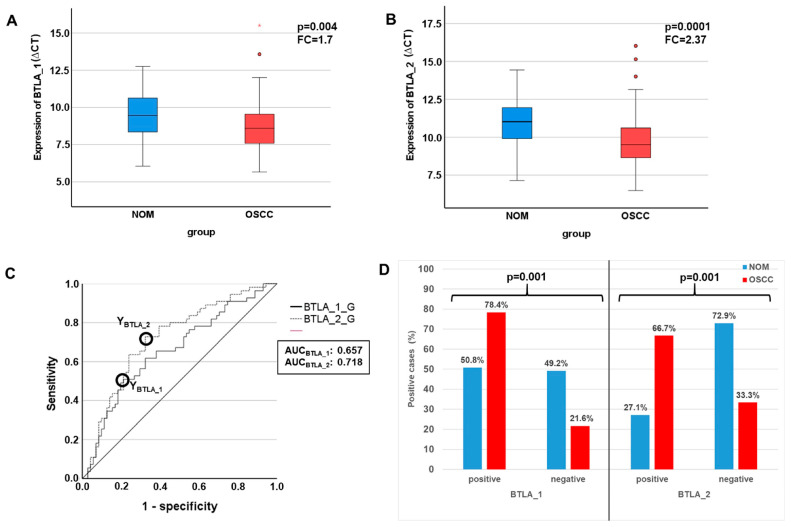
Analysis of differential expression of BTLA by RT-qPCR in OSCC compared to NOM. (**A**,**B**) Comparison of expression rates of the BTLA between OSCC and NOM group visualized by Boxplots. Expression levels are given as median ∆CT values, which means that lower values indicate a higher expression. Both BTLA transcript variants are substantially and significantly upregulated in OSCC tissues. *p*-values were calculated by MWU test. (**C**) Receiver operating characteristic curves (ROC curves) of BTLA_1 and BTLA_2 created by plotting the sensitivity (true-positive rate) against 1-specificity (false-positive rate) over all generated ∆CT values. The given AUC (area under the curve) values confirm the significant association between overexpression of the immune modulators and malignant tissues. The circles show the points of the highest Youden (Y) indices that are associated with the COP stated as a defined ∆CT value. (**D**) After division of the test and control group (OSCC and NOM group) into positive and negative subgroups based on the ascertained COPs a significant association of malignancy with expression over the COP of both BTLA isoforms was proven by χ2-test. asterisk = extreme values, Dots = outlier.

Both examined BTLA transcript variants (BTLA_1 and BTLA_2) showed significantly increased expression in OSCC compared to NOM. The mean ∆CT value of BTLA_1 in OSCC (n = 73) amounted to 8.1 and that of BTLA_2 to 9.74. In the NOM Group (n = 59) mean ∆CT was 9.46 for BTLA_1 and 10.98 for BTLA_2, respectively (Table 2, Figure 2A,B). Fold changes calculated by the ∆∆CT method revealed a prominent overexpression in the OSCC group (FC_BTLA_1_ = 1.7, FC_BTLA_2_ = 2.4; Table 2). The statistical significance of differential expression between the groups were shown by the Mann–Whitney U test (P_BTLA_1_ = 0.004, P_BTLA_2_ = 0.0001; Table 2, Figure 2A,B).

In order to confirm the statistical relevance, ROC curves (Figure 2C) were created, and the AUC was determined. The AUC value for significance of upregulation of BTLA_1 was 0.66 and that of BTLA_2 reached an AUC value of 0.72 (Table 2, Figure 2C). The sensitivity (true positive rate) reached 50.9% for BTLA_1 and 72.7% for BTLA_2. The specificity (1-false positive rate) of BLTA_1 overexpression reached 78.9% and that of BTLA_2 67.6% (Table 2). Thus, this analysis confirmed that both BTLA mRNA variants were of significant diagnostic value for discrimination between OSCC patients and healthy volunteers.

Consequently, for both transcripts the highest Youden indices, which are the optimal threshold (COP) for distinguishing two groups and are given as ∆CT values, were calculated (Table 2, Figure 2C).

Using the generated COPs, the two groups (OSCC and NOM) were subdivided into positive and negative specimens in order to confirm that these parameters allow the detection of malignancy in a certain sample. In this context, a ΔCT value lower than the COP (upregulated expression) was considered positive for malignancy. Out of the OSCC patients, 78.4% (58/74) showed increased BTLA_1 expression and 66.7% (48/72) exhibited increased BTLA_2 expression. In contrast, only 50.8% (30/59) and 27.1% (16/59) of the NOM samples showed increased BTLA_1 and BTLA _2 expression, respectively (Table 3, Figure 2D). An expression level of BTLA_1 and BTLA_2 above the COP (positivity for overexpression) was significantly associated with malignancy (*p* = 0.001; Table 3, Figure 2D). A sensitivity for detection of malignancy of 78.4% and a specificity of 49.2% were determined for BTLA_1 positivity. For BTLA_2 a sensitivity of 66.7% and a specificity of 72.9% was demonstrated (Table 3). The positive and negative predictive value of BTLA_1 and BTLA_2 expression for diagnosis of malignancy support the diagnostic value of the markers (Table 3).

The BTLA gene encodes two isoforms. BTLA_1 generates an amplicon specific for transcript variant 1 that represents the longest transcript and encodes the longest isoform [NP8614445_1]. BTLA_2 creates an amplicon of both transcript variants 1 and 2. The transcript variant lacks an alternative in-frame exon, compared to variant 1, resulting in a shorter protein (isoform 2 [NP 001078826.1] compared to isoform 1.

However, nothing is known about differences in the function of the isoforms and whether they differ in their properties and their ability to inhibit the antitumor activity of CD8+ cells. Nevertheless, the cytoplasmic region compromising the three highly conserved tyrosine motifs—the growth factor receptor-bound protein 2 (Grb2) binding site, an immunoreceptor tyrosine inhibitory motif (ITIM), and an immunoreceptor tyrosine-based switch motif (ITSM)—for function are well-kept-up. Both also carry the extracellular IgC-like domain [14]. Hence, it could be postulated, that they share similar functions.

### 2.4. Immunohistochemistry

The BTLA gene uses alternative splicing to produce two transcript variants that code for 2 isomeric proteins. A long variant and a shortened form that lacks an internal exon. A recombinant fragment within BTLA has been used as immunogenic peptide. The sequence was located within the protein between aa 1–200. This sequence lies within the sequences of the two proteins encoded by the two transcript variants. Although the exact sequence is proprietary, it could be postulated that the antibody against BTLA detects both variants equally, because both have the epitope. However, in order to confirm this, a study would have to be carried out to investigate if both isoforms were recognised in the same way. This is beyond the scope of this paper.

**Figure 3:**
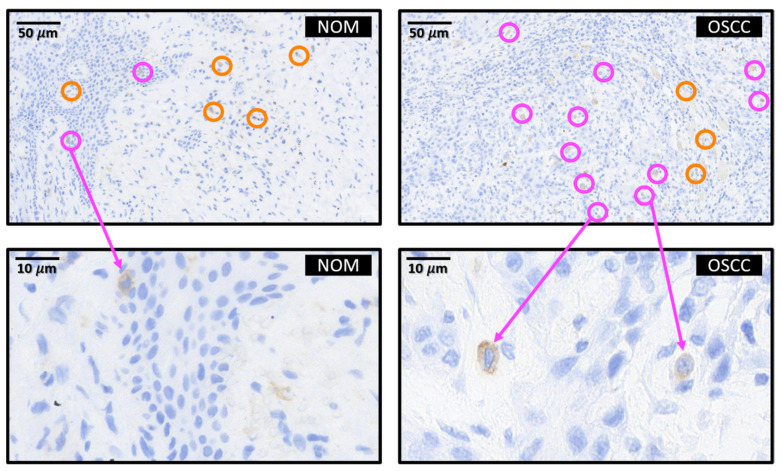
BTLA protein expression in OSCC tumor tissue (OSCC) and healthy control mucosa (NOM). Representative micrographs showing the typical expression pattern of BTLA in OSCC and NOM. For example, cells counted positive are circled in orange in the stromal compartment and circled in purple in the epithelial compartment. The respective scale and type of tissue—OSCC or NOM—is also shown in the respective tissue section. In the immunohistochemical part of the study we assessed the labelling index ((positive cells/all cells) × 100) in tumor tissue compared to healthy controls. We did not assess the staining intensity of the BTLA expressing cells. A clear allocation to lymphocytes is not possible as we did not perform multi-stainings. Morphologically, the BTLA-expressing cells are tumor-infiltrating immune cells.

Representative immunohistochemical stainings for BTLA are shown in Figure 3. We have divided the tissue into the epithelial tumor compartment and the tumor stroma. The BTLA-positive cells were counted separately. However, identification of the cell type of the BTLA-positive cells was not possible with the method used. Nevertheless, the morphology of the cells suggests that the BTLA-expressing cells are tumor-inflaming immune cells and not epithelial tumor cells. We would like to identify the lineage of the BTLA-expressing cells in future multiplex studies.

**Figure 4:**
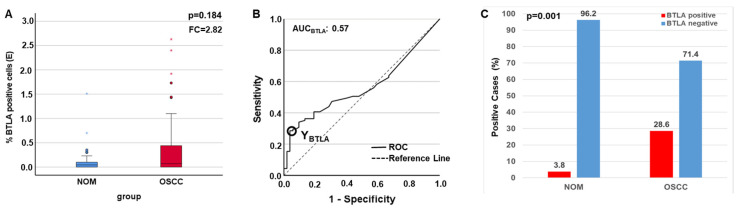
Comparison of the expression rates of BTLA in the epithelium determined by IHC. As the whole tissue is included in the analysis in PCR, this value cannot necessarily be extrapolated to the results of IHC, where only the sub-epithelium is considered. In addition, low values in RT-qPCR indicate higher expression rates, as a low CT value indicates a higher amount of mRNA and thus increased expression or transcription. In IHC, a higher percentage of LI directly indicates a higher number of positive cells [LI = (stained cells/total cells) × 100], so the differences are inverse. (**A**) Boxplots and MWU tests show a prominent epithelial overexpression of BTLA protein in the OSCC compared to the NOM. (**B**) ROC curve and AUC value indicate a significant relation between evaluated BTLA protein expression and malignant tissue. The circle shows the point of the highest Youden (Y) index that is associated to the COP stated as a definite IL value. (**C**) A significant association between OSCC diagnosis and protein expression above the COP in the epithelium was seen (χ2-test). asterisk = extreme values, Dots = outlier.

The statistic evaluation of immunohistochemical data revealed that the protein is substantially overexpressed in the epithelial part of the tumor group (mean LI_BTLA_Epithelium OSCC_ = 0.31, mean LI_BTLA_Epithelium NOM_ = 0.11, FC = 2.82), but this parameter did not reach statistical significance (*p* = 0.184) (Figure 4A, Table 4). The AUC value shows that there is only a weak discriminatory power between the two groups if the marker is used (0.57). The marker achieves low sensitivity (29%) but high specificity (96%). Therefore, the highest Youdan Index was calculated in order to generate the COP (Table 4, Figure 4B). After subdividing the two groups into positive and negative for overexpression of the protein the Chi-square test revealed that the expression above the COP (positivity for overexpression) is significantly associated with malignancy (*p* = 0.001) with a high specificity (96.2) and a high positive (PPV = 92.2) and negative predictive value (NPV = 43.5) (Table 3, Figure 4C). No differential expression could be shown in the total tissue (*p* = 0.372, FC = 1.41) and in the sub-epithelial part (*p* = 0.417, FC = −1.22) if OSCC group was compared to NOM.

### 2.5. Association of BTLA in Tissue Samples with Histomorphological and Clinical Parameters (T-, N-, UICC-Status, Grading) of OSCC Patients

Association of mRNA expression (BTLA_1, BTLA_2) and BTLA-protein expression above the COP (positivity of expression) in OSCC tissue regarding the histopathological parameters like tumor size (T1/T2), the lymph node status (grouped N0/N1), grading (1–3), UICC-status (grouped. early/late), and occurrence of a recurrence (R0; R1) was carried out by Chi-Square test. The results are summarized in Table 5. Neither the transcript variant BTLA_1 (P_T1/T2_ = 0.485, P_N0/N1_ = 0.337, P_early/late_ = 0.230, P_R0/R1_ = 0.815, P_grading_ = 0.400), nor the isoform BTLA_2 (P_T1/T2_ = 0.815, P_N0/N1_ = 0.331, P_early/late_ = 0.662, P_R0/R1_ = 0.751, P_grading_ = 0.408) was associated with any prognostic parameter. Additionally, no correlation of protein expression and any prognostic parameter could be proven. All results are summarized in Table 5.

### 2.6. Comparison of the Expression of Immune Modulators Used for Correlation Analyses in Tissue between OSCC and NOM Group

The data of the expression analysis are partly derived from previous studies [11,21,22,23]. However, the sample number was expanded in the OSCC and NOM group. In order to ensure that the key points of previous research results (PD1, PD-L1, PD-L2, and CD96) are valid for the new patient collective a statistical evaluation of the differential expression of the markers was performed. PD1 expression was significantly higher in OSCC than in NOM (*p* = 0.003; FC = 2.11). This also applies to its ligands PD-L1 (*p* = 0.001; FC = 3.31) and PD-L2 (*p* = 0.001; FC = 2.42). The statistical evaluation for CD96 expression revealed that the CD96 mRNA (*p* = 0.01 e.g., *p* = 0.001) and protein expression (p_CD96_total_ = 0.003, p_CD96_E_ = 0.011, p_CD96_S_ = 0.008) was statistically significantly increased in the OSCC group compared to NOM. The results are summarized in Table 6.

### 2.7. Spearman Correlation Analysis of BTLA to PD1 and the Ligands PD-L1 and PD-L2 and CD96

All results are summarized in Table 7.

**Figure 5:**
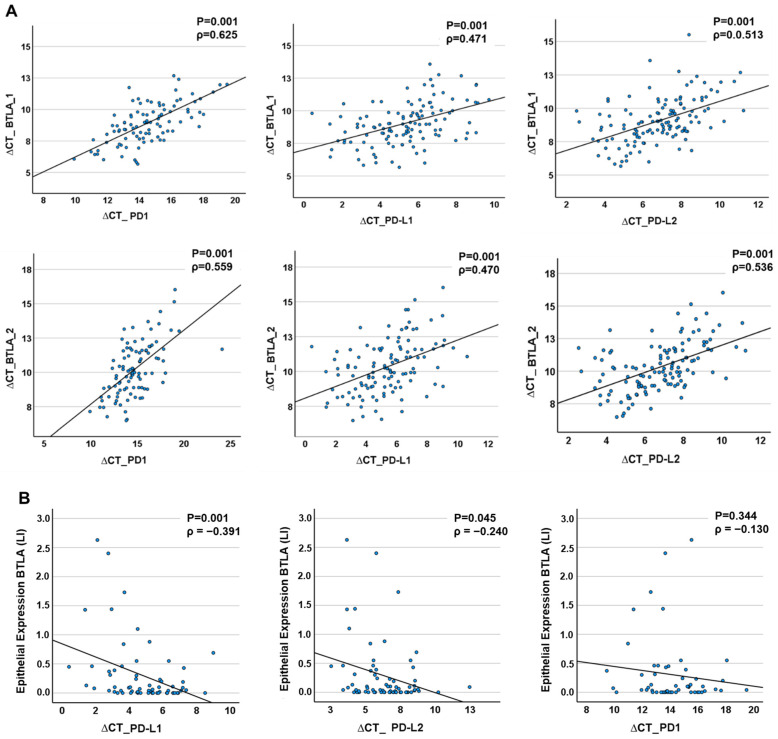
Correlation of BTLA expression to PD1 and its ligands PD-L1 and PD-L2 in tissue samples. (**A**) RT-qPCR analysis. There are positive significant correlations between the expression of the two BTLA isoforms (BTLA_1, BTLA_2) and PD1, PD-L1 and PD-L2, respectively. (**B**) IHC analysis. The Expression of the BTLA Protein correlates positively and significantly with the expression of PD1, PD-L1 und PD-L2. The *p*-value was calculated by Spearman’s rho correlation test. ρ = Correlation coefficient.

BTLA mRNA expression data in tissue specimens (OSCC and NOM) were strongly correlated with that of the PD1 and the ligands PD-L1 and PD-L2. The results are visualized in scatter plots (Figure 5). The expression of BTLA_1 mRNA and PD1, PD-L1, and PD-L2 strongly correlated positively (Spearman’s ρ PD1 = 0.625, *p* < 0.001; Spearman’s ρ PD-L1 = 0.471, *p* < 0.001; Spearman’s ρPD-L2 = 0.513, *p* < 0.0001). In addition, BTLA_2 expression showed a strong, significant positive correlation with the expression rate of all analyzed immune checkpoints (Spearman’s ρ PD1 = 0.559, *p* < 0.001, Spearman’s ρ PD-L1 = 0.470, *p* < 0.001; Spearman’s ρPD-L2 = 0.536, *p* < 0.0001). In the epithelium, a moderately positive but highly statistically significant correlation was found between BTLA protein expression and that of PD-L1 transcription (Spearman’s ρ = −0.391, *p* < 0.001). PD-L2 mRNA expression was statistically marginally significant and moderately positively correlated with BTLA protein expression (Spearman’s ρ = −0.24, *p* = 0.045). The expression of the protein correlates to lower ∆CT values. Therefore, the correlation factor ρ is negative. However, lower ∆CT values means higher mRNA transcription. Therefore, the expression of BTLA proteins is positively correlated to expression of the ligands. No significant correlation was detected between PD1 mRNA transcription levels and BTLA protein expression (Spearman’s ρ = −0.130, *p* = 0.344). The results are visualized in Figure 5.

**Figure 6:**
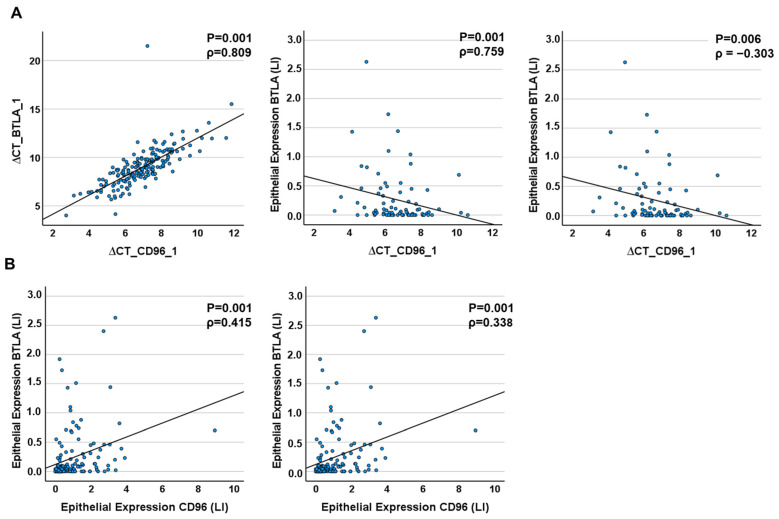
Correlation of CD96 to BTLA expression in tissue samples. (**A**) RT-qPCR analysis. There are positive significant correlations between the expression of the two BTLA isoforms (BTLA_1, BTLA_2) and CD 96 mRNA and epithelial protein expression, respectively. (**B**) IHC analysis. The Expression of the BTLA Protein correlates positively and significantly with the expression of CD96 in the epithelium and stroma of tumor tissues. The *p*-value was calculated by Spearman’s rho correlation test. ρ = Correlation coefficient.

At the RNA level, CD96 expression correlated strongly with BTLA_1 and BTLA_2 expression (for CD96_1: Spearmans´ρ_BTLA_1_ = 0.809, Spearmans´ρ_BTLA_2_ = 0.759, for CD96_3: Spearmans´ρ_BTLA_1_ = 0.794, Spearmans´ρ_BTLA_2_ = 0.814, *p* ≤ 0.001) and moderately on protein level (Table 7, Figure 6). CD96 mRNA and BTLA protein expression in total and epithelial tissue correlated weakly (Table 7, Figure 6).

Figure 5 appears to show different correlations between the expression of the immunomodulators. There is a positive correlation in Figure 5A and a negative one in Figure 5B.

The correlation informs us about the degree of correlation between two metric variables. A positive correlation means that the variables develop in the same direction. Hence, if one variable increases, this will also apply to the other variable. With a negative correlation, the opposite is true: an increase in variable 1 means a decrease in variable 2.

The discrepancy between the ratios of the values determined for the expression rates using RT-qPCR and immunohistology is due to the method used to analyse the expression. For example, low ∆CT values in RT-qPCR indicate higher expression rates, as a low CT value indicates a higher amount of mRNA and thus increased expression or transcription. In IHC, a higher percentage of LI directly indicates a higher number of positive cells [LI = (stained cells/total cells) × 100] and directly represents increased expression. In Figure 5A, the values for both BTLA and PD1, PD-L1 and PDL are determined by RT-qPCR. Here the correlation is positive because the ∆CT values decrease with increasing expression of the genes. Figure 5B compares the values from IHC with those from RT-qPCR. As higher values of LI and decreasing ∆CT values stand for increased expression, the values here are inversely correlated i.e., negative. In reality, however, the higher the expression of the protein, the higher the expression of the coding mRNA.

## 3. Discussion

In the current study a significantly increased BTLA expression could be shown in OSCC specimens compared to healthy oral mucosa at mRNA level. In addition, the expression of BTLA protein was remarkably increased in oral cancer tissues compared to healthy mucosa specimens in the epithelial compartment. However, statistical significance was not reached. At both mRNA and protein levels, there was a highly significant association between increased expression of BTLA (above a calculated cut-off point) and the diagnosis of oral cancer in a given tissue sample. This indicates that individual tissue samples can be allocated to the OSCC group or the control group depending on their BTLA expression. The relative low sensitivity and specificity show that BTLA expression alone cannot be used as reliable marker for malignancy in oral mucosa. An association of BTLA expression in tissue samples with histomorphological parameters, staging, T-status, N-status, as well as disease recurrence was not observable in the current analysis. This result is consistent with an in silico analysis performed by Yu et al. [24]. However, in that study it was shown that high BTLA expression was correlated with inferior overall survival, while there was no association between survival and PD-L1 expression [24]. Previous analyses have also failed to show a difference between PD1 [25] and PD-L1 [26] expression with respect to histomorphological parameters, although PD-L1/PD1 signalling has been shown to be a highly relevant checkpoint pathway in tumor biology. Therefore, the lack of association of BTLA expression with histomorphological and prognostic parameters does not indicate that it is not a potential immunotherapeutic target. A recent review by Andrzejczak and Karabon gives a comprehensive overview of the current understanding of BTLA biology in cancer [14]. BTLA deficient mice were shown to have higher T-cell activity and increased vulnerability towards autoimmune diseases [14]. In addition, it was demonstrated that these animals show increased number of memory-CD8^+^ T-cells [14]. These results show the supposed role of BTLA as an immunosuppressive pathway. Besides T-cells, mature B-cells also show high expression levels of BTLA. However, BTLA seems to not influence B-cell development as BTLA-deficient mice showed normal B-cell expansion [14]. In addition, a negative correlation of increasing activation with decreasing BTLA expression was found in human B-cells [14,25].

BTLA signaling was shown to lead to decreased activation of the B-cell receptor downstream signaling and decreased B-cell proliferation [14]. In dendritic cells (DCs), BTLA overexpression was associated with reduced maturation leading to immune tolerance [27]. In addition, it was shown that BTLA signaling can suppress the proliferation of DCs indicating BTLA also being a suppressive checkpoint for DCs [26]. These data point out that BTLA expression of various immune cells is associated with a more immunosuppressive phenotype.

In the context of malignant tumors, upregulated BTLA expression is linked to the inhibition of anti-tumor immunity and inferior prognosis [14]. In CLL, BTLA expression as well as the number of BTLA positive NK-cells were increased [14]. In addition, higher BTLA mRNA levels were found in B- and T-cells compared to healthy controls [25].

In an immunohistochemical analysis in human lung cancer samples, an association of BTLA expression, shorter survival and lymphatic invasion was found [28]. In addition, BTLA expression and PD-L1 expression correlated strongly in a positive manner [28]. Results of the current study also show a significant positive correlation of BTLA expression to the immune checkpoints of the PD axis at the mRNA level, and except for PD1, also on protein level.

A flow cytometric analysis in lung cancer samples revealed that BTLA expressing tumor infiltrating CD8 positive T-cells also showed increased expression of the checkpoint receptors PD1, CTLA4, LAG-3 and TIM-3 [29]. Based on the checkpoint expression profiles of different T-cell populations, the authors conclude that BTLA expression is a marker for late-stage T-cell exhaustion [29]. These results support the concept of an increased immunosuppressive state also present in the TME of OSCC. In contrast, in melanoma treated with adoptive cell therapy, BTLA expressing CD8 positive cells were found to be associated with a positive treatment outcome [14]. An in vitro co-culture experiment with melanoma cells and T-cells showed that the BTLA ligand HVEM were expressed on all analyzed human melanoma cell lines [30]. The simultaneous application of an anti-BTLA antibody led to an increase of the T-cell/melanoma cell ratio indicating an immune activating and anti-tumoral activity of BTLA inhibition [30].

In gastric cancer, a significantly increased BTLA expression was seen compared to dysplastic and metaplastic gastric cancer precursor lesions in an mRNA analysis. On protein level analyzed by immunohistochemistry, BTLA expression in metaplastic and gastric cancer lesions was significantly increased compared to dysplastic lesions [31]. In addition, advanced stage gastric cancer showed significantly higher expression levels compared to early-stage malignancies [31]. The increased expression of BTLA in malignant tissue is consistent with the results of the current study analyzing OSCC samples. However, we did not detect differences in BTLA expression in advanced-stage tumors compared to early-stage malignancies.

Recently, in addition to the immune checkpoint receptors and ligands on the cell membrane, a number of soluble immune checkpoints have been identified and their plasma levels measured [14]. These include sPD-L1, sPD1, sCTLA4, sCD80, sTIM3, sLAG3, sB7-H3, sBTLA and sHVEM. These checkpoints are generated by alternative splicing or proteolytic processes and play an important role in immune regulation, are involved in the development and prognosis of cancer and are considered potential biomarkers and therapeutic targets [32]. They are also ascribed similar functions to the membrane-bound forms. Such forms also exist in OSCC, including BTLA. A high concentration of sBTLA could be a prognostic and predictive marker for response to treatment with immune checkpoint inhibitors. The role of sBTLA in carcinogenesis has not yet been clarified [14]. BTLA lacks the transmembrane domain. Otherwise, this transcript variant does not differ from the other isoforms. It is therefore not possible to construct primers for RT-qPCR that exclusively detect this soluble variant. In order to be able to detect differential gene expression, other methods such as multiplex immunoassays would have to be used. This should be the subject of future investigations.

With the BTLA-inhibitor Tifcemalimab being in early clinical trials in humans, a therapeutic targeting of BTLA is in clinical reach [14]. First results show good safety and potentially clinical efficiency. HVEM and PD-L1 expression were shown to be potential biomarkers for therapy efficiency [14]. In addition, a phase III study with a total of 756 participants is planned to test the clinical efficiency of combined anti-BTLA and anti-PD1 ICI therapy as adjuvant treatment for early-stage small cell lung cancer after initial radiochemotherapy (NCT06095583). This and further studies will show if anti-BTLA ICI therapy is clinically efficient [33].

An in silico analysis performed using the Cancer Genome Atlas database searched for correlations between PD-L1 expression and other immune checkpoint genes in OSCC [24]. A positive correlation between PD-L1 and BTLA was detected supporting the possibility of combination therapies [24]. These results are consistent with our current data that could show a significant positive correlation between BTLA and PD-L1 expression on mRNA and protein-level. Additionally, PD1 and BTLA act through SCR-homologous phosphatases, leading to loss of T-cell function, extinction and T-cell exhaustion [14,34]. Immune checkpoint inhibition reverses this effect. It is therefore conceivable that with increased expression of both checkpoints, the effect of one inhibitor is counteracted by the other ICP, ultimately leading to the maintenance of immunosuppression via this mechanism. The simultaneous application of antibodies against BTLA and PD1 can overcome the inhibition in the case of overexpression of both ICPs, thus increasing the response rate and preventing adaptive immune tolerance. The significant co-expression and strong correlation of immune checkpoint expression observed in our study suggests the possibility that the effect of one ICI could be neutralized by the other and that this problem could also be overcome by combining the antibodies. This and our results support a combination of immune checkpoint therapies targeting BTLA and PD axis in OSCC.

Immunotherapy has become increasingly important in recent years, particularly with the development of immune checkpoint inhibitors targeting PD1/PD-L1 or BTLA [35]. These therapy options have proven to be effective in many types of cancer by stimulating the immune system to attack tumor cells—also in head and neck cancer. However, there are also challenges such as primary resistance and acquired resistance that can limit the effectiveness of these therapies. Therefore, the search for diagnostic markers that can predict response to therapy and the development of combination therapies is gaining importance. In this context, immune checkpoint inhibitors such as CD96 also play an important role. The checkpoint inhibitor CD96 is currently tested in phase I and II studies as monotherapy and in combination with PD1-inhibition in advanced solid malignancies and lymphomas [14]. Wang et al. showed that CD96 and PD1 are co-operative and negatively regulate the function of tumor infiltrating lymphocytes [36]. Blockade of BTLA and CD96 is promising for use in combination with PD1 blockade in the treatment of cervical cancer, as targeting the ICI CD96 overcomes PD1 blockade resistance by boosting CD8+ T-cell function.

Results of the current study showed an increased BTLA, CD96 and PD1 expression in cancer tissue samples and a significant positive correlation of BTLA and PD1 expression to the immune checkpoint CD96. This indicates that multiple immune checkpoint pathways in oral mucosa and OSCC tissue are co-regulated. Hence, the data highlight the expression of BTLA in oral cancer and motivate clinical trials of anti-BTLA therapy—preferentially as a concept of a simultaneous therapeutic targeting of multiple inhibitory signalling pathways like PD1, CD96 and BTLA—in oral cancer.

Overall, the findings of this study provide valuable insights into the role of BTLA in OSCC and its potential as a therapeutic target. Further research is needed to explore the mechanisms underlying the co-regulation of immune checkpoint pathways and the potential synergistic effects of targeting multiple pathways in oral cancer treatment. Clinical trials investigating the efficacy of anti-BTLA therapy, either alone or in combination with other immune checkpoint inhibitors, are warranted to evaluate its potential as a novel treatment approach for OSCC patients.

## 4. Material and Methods

### 4.1. Description of the Study Collective and Collection of Samples

The patient collective studied included a total of 207 samples divided into 2 groups. Group 1 (OSCC Group) comprises 102 patients with an initial diagnosis of oral squamous cell carcinoma and were not treated prior to study inclusion. Tissue of the oral mucosa from 105 healthy volunteers with normal oral mucosa (NOM group, group 2) served as the comparison group. All samples were taken in the period from April 2010 to January 2020 at the Department of Oral and Cranio-Maxillofacial Surgery of the Friedrich-Alexander University Erlangen-Nürnberg. The study was performed in accordance with the Declaration of Helsinki. A positive vote of the local ethics committee is available (ethics application number: 3962, date: 16 April 2009, prolongation: 1 December 2010; Preparation of a new follow-up application 2019 (application number 415_20 B)). Healthy volunteers (NOM group) were chosen based on the absence of inflammation and any malignant disease. OSCC tissues were classified in regard to their grading (G1–G3; differentiation status), the clinical UICC-stage (I–IV) and TNM classification according to the guidelines of the World Health Organization classification (2017) of tumors of the head and neck and the International Union Against Cancer [37,38]. Afterwards, lymph node status was grouped according to absence (N0) or presence (N+) of lymph node metastases. Additionally, clinical stages were grouped as early (stage I and II) and late (stage III and IV) stages and, based on tumor size divided into small (T1 and T2) and large (T3 and T4) tumors. All clinical and histopathological parameters are given in Table 1.

### 4.2. Sampling of Tissue Specimens

Tissue samples of NOM group were acquired during minor routinely done surgeries. These control cases are patients that were healthy regarding inflammatory or malignant diseases of the oral cavity and had no major systemic diseases. These patients received minor oral surgery like wisdom tooth removal or removal of teeth due to orthodontic treatment. During this procedure, small tissue samples were removed. None of the healthy controls had oral leukoplakia and none of them was suspected of having OSCC.

Tumor specimens of the OSCC group were gained during tumor resection. Each sample was divided into two pieces. One was used for histological examination; the second was immediately transferred into RNAlater^®^ (Qiagen, Hilden, Germany) and were fixed by incubation at 4 °C for at least 24 h. Afterwards they were stored at −80 °C until mRNA isolation. The tumor samples to be analyzed contained at least 70–80% malignant epithelial tissue.

The localizations of the biopsies are different. We consider the immune environment in the oral mucosa as relatively homogenous. Additionally, considering different anatomical sites would make the subgroups extremely small. Therefore, we pooled all samples obtained from the oral mucosa.

### 4.3. Isolation of mRNA and RT-qPCR Analysis

For RNA isolation from RNAlater^®^ samples, initially a tissue disruption using a Precellys^®^ (Bertin Instruments Company, Bertin, France) was performed and total RNA was isolated by the use of the Qiagen “miRNeasy mini-Kit” (Qiagen Company, Hilden, Germany) according to the manufacturer’s instructions.

To determine the quality and quantity of the RNA samples, their concentrations were determined using the Nano-Drop 3.3 ND1000 spectrophotometer and the corresponding ND-1000 software (Thermo Fisher Scientific Company in Waltham, MA, USA).

Reverse Transcription of RNA into cDNA was done using the Transcriptor High-Fidelity cDNA Synthesis Kit according to the manufacturer’s recommendations (Roche, Mannheim, Germany).

For semi-quantitative analysis of BTLA expression gene-specific primers (Table 8) and the QuantiTect SYBR^®^ Green PCR Kit (Qiagen, Hilden, Germany) were used. For correlation analysis, BTLA expression was compared with the previously published PD1, PD-L1, PD-L2 and CD96 expression (Table 8) [11,21,22,23]. The annealing temperature for all primer pairs was 60 °C. Number of cycles totaled to 40. The BTLA gene encodes two isoforms. The transcript variant 1 represents the longer transcript and encodes the longer isoform. The transcript variant 2 lacks an alternate in-frame exon, compared to variant 1, resulting in a shorter protein (isoform 2), compared to isoform 1. For BTLA two primer sets were applied. The primer set of BTLA_2 was extracted from the sequence of the transcript variant 2. It is located outside of the sequence of the alternate in-frame exon. Thus, this pair allows the amplification of a gene specific amplicon of both isoforms. Amplicons that are exclusively generated by the long transcript variant are not created. On the other hand, the primer set BTLA_1 makes only products based on the long transcript variant, which means that only the long isoform 1 is detected.

Data acquisition and analysis was made using the ABI Prism 7300 of Applied Biosystems (ThermoFisher Scientific Inc., Waltham, MA, USA). The data were normalized by the ∆CT method using GAPDH as an internal control. The relative quantification of differences in gene expression between the two groups was based on the ∆∆CT-method. (RQ = Fold change (FC) = 2^−∆∆CT^).

The method is described in more detail below. ∆CT values were calculated as follows: ∆CT (sample) = average CT (target gene) − average CT (GAPDH; endogenous control). The average ∆CT value was then calculated for each target gene in the groups: Average ∆CT (group) = sum of all ∆CT/number of all samples in the group. In order to compare the groups with respect to the expression of the target gene, after calculating the ∆∆CT value (∆∆CT = ∆CT control − ∆CT test group), the relative change in expression (RQ), which corresponds to the fold change (FC), was calculated as follows: RQ/FC = 2^−∆∆CT^. If the fold change is greater than ±1.5, the analyzed sample is overexpressed or underexpressed compared to the control sample. If the value is smaller or equal to ±1.5, there is no differential expression.

### 4.4. Detection and Quantitative Analysis of BLTA and CD96 Expression by Immunohistochemistry

Serial 2 µm thick sections were prepared from the formalin-fixed and paraffin-embedded tissue samples. To unmask the epitope, pretreatment was performed in EDTA buffer (pH 9.0, vitro master diagnostica, Sevilla, Spain) at 100 °C for 20 min. The Anti-BTLA antibody (dilution 1:500, EPR22224-27ab230976, Abcam, Berlin, Germany) or antibody against CD96 (Abcam, ab264416, GR13312304-3, dilution 1:200) was applied. Staining was done using the Bridge Vision Kit according to the manufacturer’s recommendation (ImmunoLogic, Duiven, The Netherlands). Tonsillar tissue served as positive control. Membranous staining was defined as positive result. All specimens were digitalized by full scanning and 40× magnification using the Pannoramic 250 Flash III scanner and Pannoramic Viewer 1.15.2 software (3DHISTECH^®^, Budapest, Hungary). For each scanned tissue sample, three same sized image fields (Regions of Interest, ROIs) were created in Case Viewer 2.3 software (3DHISTECH^®^, Budapest, Hungary). Those ROIs were divided into an epithelial and subepithelial compartment and all compartments were exported into TIF format to be analyzed via QuPath 0.4.1. The overall cell count was automatically done using QuPath, the positive cell count was manually done via Case Viewer. For each ROI-compartment (epithelium, stroma and overall) the ratio of positive cells to the total number of cells was determined. With the program QuPath we were able to do an automatic cell count, without performing a labeling index. Calculating the labeling index was done manually.

The described labeling index is calculated as follows via the formula:

(positive cells/total number of cells) × 100.

This allows conclusions to be drawn about the ratio of positive cells to total cells. This procedure was done for 3 selected regions (ROIs), from which the average value was formed. The labeling index (LI) values given are percentage values. A value below 1 therefore corresponds to a percentage below 1%. This means that the number of cells tested positive is multiple magnitudes smaller than the total cells counted in the same ROI.

Afterwards, the mean value was calculated. This percentage values were used in the statistical expression analysis, now called labelling index (LI).

### 4.5. Statistics

For evaluation of the results, the statistical software package SPSS 23 (SPSS Inc., Chicago, IL, USA) was used. It is based on the data collected of ∆CT data generated by RT-qPCR and the IL gained by IHC. Prior to the statistical analysis, the data were tested for their normal distribution by utilizing the Shapiro–Wilk test.

Box-whisker plots visualize the expression differences by displaying the median, interquartile range, and minimum and maximum values of the gene expression in the different groups.

Nonparametric tests were used because the data are not normally distributed. Kruskal–Wallis test and Mann–Whitney U Test (MWU test) were done to decide whether the expressions between the groups differ significantly in the expression levels of the genes. A *p*-value ≤ 0.05 was considered statistically significant.

In qPCR analysis the relative gene expression (fold change, FC; RQ) between groups were calculated using the ∆∆Ct-method. The FC in immunohistological staining corresponded to the ratio of mean IL of the groups (LI_OSCC_/LI_NOM_). Values superior to 2 were regarded as relevantly increased.

To determine a Cut-Off Point (COP), a Receiver Operator Characteristic (ROC) curve is created. This is done by plotting the sensitivity against the specificity (1-specificity). The area under the curve shows how well each marker can discriminate between the two groups. The Youden indices (Y = sensitivity − (1-specificity)) are then calculated from the ROC curve. The COP is derived from the highest Youden index (Y = maximum possible sensitivity and specificity on the curve). The Y, and therefore the COP, correspond to a specific ∆CT value. The individual patient samples can then be divided into 2 subgroups. All values greater than the COP are marked as negative and all values less than the COP are marked as positive (overexpression of the biomarker). This allows dividing a collective into two subgroups showing either underexpression (negative) or overexpression (positive) values with respect to COP. After this sub-classification, the Chi-square test (χ2-test) was used to explore whether the overexpression of the immune checkpoint is associated with diagnosis, TNM classification of OSCC and Grading. A statistical significance is set at a *p*-value ≤ 0.05. Lastly, the positive (PPV) and negative predictive values (NPV) were calculated.

Correlation analyses of mRNA (two variants of BTLA named BTLA_1 and BTLA_2) and protein expression of BTLA with the expression of different immune markers were done by Spearman’s correlation. A *p* value < 0.05 was considered to be significant. ρ was interpreted according to Cohen et al. [39].

The correlation informs us about the degree of correlation between two metric variables. A positive correlation means that the variables develop in the same direction. Hence, if one variable increases, this will also apply to the other variable. With a negative correlation, the opposite is true: an increase in variable 1 means a decrease in variable 2. A correlation consists of a correlation coefficient and a *p*-value. There are different correlation coefficients that are used for different data: Pearson correlation coefficient or the Spearman correlation coefficient. If the data is not normally distributed and/or the correlation is not linear, the Spearman correlation will be used. The correlation coefficient indicates the strength and direction of the correlation. It lies between −1 and 1. A value close to −1 indicates a strong negative correlation. A value close to 1 indicates a strong positive correlation. There is no correlation if the value is close to 0. Interpretation was done according to Cohen et al. (ρ = 0.10 weakly, ρ = 0.3 moderately, ρ = 0.5 strongly correlated). The *p*-value indicates whether the correlation coefficient differs significantly from 0, i.e., whether there is a significant correlation. In most cases, *p*-values smaller than 0.05 are described as statistically significant [39]. The appropriate figure is a scatter diagram that visualises the correlation.

## 5. Conclusions

BTLA is an emerging therapeutic target for immunotherapy in human cancer. This analysis in human OSCC tissue samples gives a first overview of alterations of BTLA expression on mRNA- and protein-level. The results of the current study show that BTLA expression is upregulated in oral cancer. The significant positive correlation between BTLA expression and other immune checkpoints including CD96 and PD1 with its ligands indicate that BTLA-inhibition in oral cancer might be promising, especially in the form of a combination immunotherapy. These data motivate further studies to clarify the exact cellular source of BTLA expression in healthy mucosa and oral cancer as well as functional analyses. Currently initiated therapy studies in other solid malignancies will show the therapeutic value of BTLA inhibition in the future.

## Figures and Tables

**Table 1:** Number of cases, demographic and clinical data of the studied patient collective. For the OSCC patients, staging parameters (T-, N-, L-status, grading, clinical UICC stage) and occurrence of recurrence are shown.

		Patients (OSCC)		Healthy Volunteers (NOM)	
		n	% of Cases	n	% of Cases
**Number of cases**		102		105	
**Gender**	Male	70	68.6	66	62.9
***p* = 0.233**	Female	32	31.4	39	37.1
**Mean age ± SD**		62.57 ± 11.92 years		48.62 ± 20.18 years	
***p* = 0.001**					
**Range of age**		31–93 years		18–88 years	
			
			**Valid cases %**		
Tumor Status *	T1/T2	57	58.8		
	T3/T4	40	41.2		
	Unknown	5			
N-Status *	N0	56	57.1		
	N1	42	42.9		
	Unknown	4			
**Grading**	G1	9	9.4		
	G2	45	46.9		
	G3	42	43.8		
	Unknown	6			
Clinical stage *	Early	40	44.4		
	Late	50	55.6		
	Unknown	12			
**Recurrence**	No	77	80.2		
	Yes	19	19.8		
	Unknown	6			

* = the prognostic parameters are grouped: Tumor size (small ones = T1/T2, large ones (T3/T4), N-status (N0 = without lymph node involvement, N1 = lymph node involvement), Clinical stage (early = stage 1 and 2, late = stage 3 and 4).

**Table 2:** Determination of the expression levels of BTLA in the tissues by RT-qPCR analyses, comparison of the expression between the OSCC and NOM group and determination of the statistical significance of the expression differences.

Group	N	∆CT Mean Value	SD	AUC-Value	Y	COP	SEN %	SPE %	FC	*p* Value
Expression Analyses by RT-qPCR (∆CT)										
BTLA_1										
OSCC	73	8.1	1.73							
NOM	59	9.46	1.566	0.66	0.30	9.59	50.9	78.9	1.68	0.004
BTLA_2										
OSCC	71	9.74	1.85							
NOM	59	10.98	1.63	0.72	0.40	10.40	72.7	67.6	2.37	0.0001

Higher average ∆CT values indicate lower expression of the gene. Both transcript variants of BTLA are significantly increased in the OSCC group. N = Number of cases, SD = Standard Deviation, AUC = Area Under the Curve, Y = Youden index; COP = Cut Off Point, SEN = Sensitivity and SPE = Specificity for distinction of the two groups; FC = Fold Change = relative change in expression level between groups, *p*-value provided by MWU test.

**Table 3:** Association between diagnosis and positivity of BTLA expression in a specific tissue.

	N	Positive N/%	Negative N/%	*p*-Value	SEN %	SPE %	PPV %	NPV %
**BTLA_1**								
OSCC	74	58/78.4	16/21.6					
NOM	59	30/50.8	29/49.2	**<0.001**	78.4	49.2	65.9	64.4
**BTLA_2**								
OSCC	72	48/66.7	24/33.3					
NOM	59	16/27.1	43/72.9	**<0.001**	66.7	72.9	75	64.2
BTLA_IHC *								
OSCC	91	26/28.6	65/71.4					
NOM	52	2/3.8	50/96.2	**<0.001**	28.6	96.2	92.2	43.5

A highly statistically relevant association between expression of BTLA above the calculated threshold (COP) and malignancy was demonstrated for both mRNA and epithelial protein overexpression. N = number of cases; SEN = Sensitivity and SPE = Specificity for diagnostic use between groups, *p*-value by χ2-test; PPV = Positive Prediction Value; PPN = Negative Prediction Value. * total tissue.

**Table 4:** Determination of the expression levels of BTLA in the tissues by immunohistochemical analyses, comparison of the expression between the OSCC and NOM group and determination of the statistical significance of the expression differences.

Group	N	LI Mean Value	SD	AUC-Value	Y	COP	SEN %	SPE %	FC	*p* Value
BTLA_T										
PECM	91	0.41	0.49							
OSCC	52	0.29	0.41	0.53	n.d.	n.d.	n.d.	n.d.	1.41	0.372
BTLA_E										
OSCC	91	0.31	0.52							
NOM	52	0.11	0.23	0.57	0.25	0.36	29	96	2.82	0.184
BTLA_S										
OSCC	81	0.66	0.65							
NOM	52	0.54	0.79	0.46	n.d.	n.d.	n.d.	n.d.	1.22	0.417

Prominent increased expression could be shown in the epithelial part of the tumor tissue. However, no significance was achieved. The Labeling Index (LI) values given are percentage values. A value below 1 therefore corresponds to a percentage below 1%. This means that the number of cells tested positive is multiple magnitudes smaller than the total cells counted in the same ROI. N = Number of cases. T = Total/whole tissue, E = Epithelial, S = stromal, LI = Labelling Index, SD = Standard Deviation, AUC = Area Under the Curve, Y = Youden Index; COP = Cut Off Point, SEN = Sensitivity and SPE = Specificity for distinction of the two groups; FC = Fold Change = relative change in expression level between groups, *p*-value provided by MWU test. n.d. = not determined

**Table 5:** Relationship between overexpression of BTLA and clinical and histopathological parameters.

Gene *Parameter*	N	Average Expression	SD	FC	*p*-Value * MWU	*p*-Value ** χ2
**BTLA_1**						
***Tumor size***						
**T1/T2**	39	8.656	1.707	0.88	0.594	0.485
**T3/T4**	31	8.475	1.352			
***N-Status***						
**N0**	37	8.712	1.598	0.83	0.479	0.337
**N1**	34	8.448	1.492			
***UICC-Status***						
**early**	28	8.790	1.734	1.28	0.382	0.230
**late**	37	8.430	1.49			
***Grading***						
**G1**	5	8.862	0.287	n.d.	0.438	0.400
**G2**	36	8.588	1.476			
**G3**	29	8.542	1.78			
***Recurrence***						
**R0**	53	8.59	1.841	1.08	0.541	0.815
**R1**	16	8.695	1.981			
**BTLA_2**						
***Tumor size***						
**T1/T2**	37	9.536	1.713	1.15	0.754	0.815
**T3/T4**	31	9.732	1.812			
***N-Status***						
**N0**	36	9.558	1.57	1.11	0.933	0.331
**N1**	34	9.704	1.913			
***UICC-Status***						
**early**	26	9.636	1.706	1.01	0.812	0.662
**late**	37	9.626	1.880			
***Grading***						
**G1**	5	9.729	0.214	n.d.	0.821	0.408
**G2**	35	9.484	1.541			
**G3**	29	9.793	2.088			
***Recurrence***						
**R0**	51	9.665	1.540	1.05	0.617	0.751
**R1**	16	9.732	2.521			
BTLA protein						
***Tumor size***						
**T1/T2**	46	0.297	0.402	1.12	0.118	0.428
**T3/T4**	34	0.334	0.611			
***N-Status***						
**N0**	44	0.276	0.402	1.33	0.944	0.653
**N1**	35	0.368	0.604			
***UICC-Status***						
**early**	28	0.232	0.269	1.599	0.496	0.984
**late**	47	0.371	0.607			
***Grading***						
**G1**	5	0.154	0.178	n.d.	0.919	0.780
**G2**	38	0.339	0.523			
**G3**	31	0.338	0.538			
***Recurrence***						
**R0**	52	0.317	0.481	0.82	0.451	0.313
**R1**	22	0.260	0.475			

Statistical evaluation of differential expression in OSCC tissues of different prognostic groups was performed using the Mann–Whitney U * and Kruskal–Wallis test concerning grading. To verify whether expression above the COP was significantly related to given parameters in certain tissues, the Chi-square test ** was performed.

**Table 6:** Summary of the statistical evaluation of the differential expression of the immune modulators whose expression rates were correlated to that of BTLA in OSCC tissue compared to NOM.

Target	N	AUC-Value	FC	*p*-Value
PD1 *^$^	160	0.67	**2.11**	**0.003**
PD-L1 *^$^	175	0.77	**3.31**	**≤0.001**
PD-L2 *^$^	175	0.78	**2.42**	**≤0.001**
CD96_1 *^$^	185	0.65	1.28	**0.01**
CD96_2 *^$^	185	0.65	1.56	**≤0.001**
CD96_G *^§^	141	0.65	1.53	**0.003**
CD96_E *^§^	141	0.63	1.28	**0.011**
CD96_S *^§^	141	0.64	1.39	**0.008**

The expression of mRNA of all targets is significantly increased in OSCC tissues. * Data of CD 96, PD1 and its ligands are partly published. AUC = area under the curve, FC = Fold Change = Relative change in expression level between groups, *p*-value provided by MWU test. G = Total, E = Epithelial, S = stromal tissue. ^$^ = PCR, ^§^ = IHC.

**Table 7:** Correlation analysis of the expression of the examined immune checkpoints.

		PCR	IHC BTLA			
		BTLA_1	BLTLA_2	T	E	S
PCR						
CD96_1	Spearman’s ρ	**0.809**	**0.759**	***−0.289***	***−0.303***	−0.155
	*p*-value	**<0.001**	**<0.001**	**0.009**	**0.006**	0.190
	N	178	181	80	80	73
CD96_3	Spearman´s ρ	**0.794**	**0.814**	−0.229	−0.229	−0.061
	*p*-value	**<0.001**	**<0.001**	**0.041**	**0.007**	0.610
	N	177	181	80	80	73
PD1	Spearman´s ρ	**0.625**	**0.559**	−0.05	−0.103	−0.020
	*p*-value	**<0.001**	**<0.001**	0.725	0.344	0.872
	N	170	173	78	78	71
PD-L1	Spearman´s ρ	***0.471***	***0.470***	***−0.333***	***−0.391***	−0.099
	*p*-value	**<0.001**	**<0.001**	**0.006**	**<0.001**	0.410
	N	173	176	**79**	**79**	72
PD-L2	Spearman´s ρ	**0.513**	**0.536**	−0.277	−0.240	−0.111
	*p*-value	**<0.001**	**<0.001**	**0.02**	**0.045**	0.346
	N	175	178	81	81	74
IHC						
CD96_Tl	Spearman´s ρ	−0.132	−0.214	***0.417***	***0.364***	***0.247***
	*p*-value	0.249	0.057	**<0.001**	**<0.001**	**0.004**
	N	78	80	140	140	131
CD96_E	Spearman´s ρ	−0.100	−0.154	***0.300***	**0.415**	0.09
	*p*-value	0.379	0.171	**<0.001**	**<0.001**	0.292
	N	79	81	141	141	131
CD96_S	Spearman´s ρ	−0.081	−0.144	**0.496**	***0.338***	***0.424***
	*p*-value	0.493	0.325	**<0.001**	**<0.001**	**<0.001**
	N	74	76	135	135	130
BTLA_T	Spearman´s ρ	−0.196	−0.305	-	**0.635**	**0.804**
	*p*-value	0.086	***0.006***		**<0.001**	**<0.001**
	N	78	***80***		**143**	**133**
BTLA_E	Spearman´s ρ	−0.191	−0.220	**0.635**	-	***0.292***
	*p*-value	0.094	0.050	**<0.001**		**<0.001**
	N	78	80	143		133
BTLA_S	Spearman´s ρ	−0.150	−0.208	**0.804**	***0.292***	-
	*p*-value	0.212	0.078	**<0.001**	**<0.001**	
	N	71	73	133	133	

The Table shows the correlation between the expression of the transcript variants of BTLA (1, 2) and BTLA protein with the expression of the immune modulators PD1, PD-L1, PD-L2, CD96 (mRNA and protein). Statistically relevant *p* values and strong spearman correlation factors (ρ) are printed in bold. Moderate ρ are in bold italics. N = number of cases.

**Table 8:** The selected primers for RT-qPCR expression analyses of the investigated target gens.

Primer	Sequence (5′ to 3′)	Primer (bp)	Amplicon (bp)	Accession Number
**BTLA_1/s**	ACAATGGGTCATACCGCTGTT	21		NM_181780.2 ^#^
**BTLA_1/as**	CTTGGAGGGTCGTTCTGAGG	20	110	
**BTLA_2/s**	CTCTGACACAGCAGGAAGGG	20		NM_001085357.2 ^##^
**BTLA_2/as**	TTTTGCCTGGTGCTTGCTTC	20	81	
**PD1/s**	AAACCCTGGTGGTTGGTGTC	20		NM_005018.2
**PD1/as**	CTCCTATTGTCCCTCGTGCG	20	105	
**PD-L2/s**	ACAGTGCTATCTGAACCTGTGG	22		NM_025239.3
**PD-L2/as**	CTGCAGGCCACCGAATTCTT	20	98	
**CD96_1 s**	ACCTCCAGTGGGACAGATACC	21		NM_198196.3 *
**CD96-1 as**	GAAGTGTTGAGCCTGCACCT	20	91	
**CD96_3 s**	GCATGGTCGGTGGAGGATAA	20		NM_001318889 **
**CD96_3 as**	GGACTGGAGAGAGGTGGAGT	20	130	
**GAPDH/s**	GACCCCTTCATTGACCTCAACTA	23		NM_002046.5
**GAPDH/as**	GAATTTGCCATGGGTGGAAT	20	102	

The annealing temperature for every amplification analysis is 60 °C. Number of cycles totaled to 40. The BTLA gene encodes two isoforms. BTLA_1 generates an amplicon specific for transcript variant 1 ^#^ that represents the longest transcript and encodes the longest isoform. BTLA_2 creates an amplicon of both transcript variants 1 and 2 ^##^. The transcript variant 2 ^##^ lacks an alternate in-frame exon, compared to variant 1, resulting in a shorter protein (isoform 2) compared to isoform 1. §Specific amplification of sequences of transcript variant 1 and 4, simultaneously. Four transcript variants exist for CD96. One of these, variant 4 is a non-coding RNA. Hence, three different protein isoforms are encoded. Variant 1 codes the longest isoform (NP_001305818.1). Compared to variant 1, variant 2 lacks an alternate in-frame exon resulting in a shorter protein (isoform 2; NP_937839). Variant 3 encodes isoform 3 (NP_005807.1) which is shorter and has a distinct C-terminus, compared to isoform 1, because it lacks several exons, and its 3′ terminal exon extends past a splice site that is used in variant 1. This results in a novel 3′ coding region and 3′ UTR, compared to isoform 1. * Amplification of transcript variant 1 of CD96 that encodes the longer isoform (detection of all transcript variants except for transcript variant 3). ** Amplification of a sequence of transcript variant 3 of CD96 that encodes a shorter isoform with a distinct C-terminus compared to variant 1.

## Data Availability

The original contributions presented in the study are included in the article, further inquiries can be directed to the corresponding author.
